# Lubo Airway Collar as a Rescue in Magnetic Resonance Imaging

**DOI:** 10.7759/cureus.27063

**Published:** 2022-07-20

**Authors:** Balaji Vaithialingam, Abhishek Kotwal

**Affiliations:** 1 Department of Neuroanaesthesia and Neurocritical Care, National Institute of Mental Health and Neurosciences, Bengaluru, IND; 2 Department of Neuroimaging and Interventional Radiology, National Institute of Mental Health and Neurosciences, Bengaluru, IND

**Keywords:** jaw thrust, anesthesia, magnetic resonance imaging, lubo collar, upper airway obstruction

## Abstract

Adverse respiratory events in the out-of-operation theatre premises can be challenging to anesthesiologists. Manual jaw thrust application by anesthesiologists can be a temporary solution during an adverse airway event in remote locations. This report highlights the utility of a novel collar in alleviating upper airway obstruction in a patient in the MRI suite under IV sedation. The Lubo collar provides an automated jaw thrust and can also be applied electively for patients undergoing MRI under sedation and who are at risk of upper airway obstruction.

## Introduction

The loss of upper airway muscle tone is common with administering anesthetic agents [1]. Therefore, the application of manual jaw thrust is imperative in an adverse respiratory event to maintain the upper airway patency while conducting MRI under IV sedation. The Lubo collar (Inovytec Medical Solutions, Israel) can simultaneously provide automated jaw thrust and cervical spine control. We report the successful use of the MRI-compatible Lubo collar in a patient with upper airway obstruction and rapid oxygen desaturation in the MRI suite.

## Case presentation

**Figure 1:**
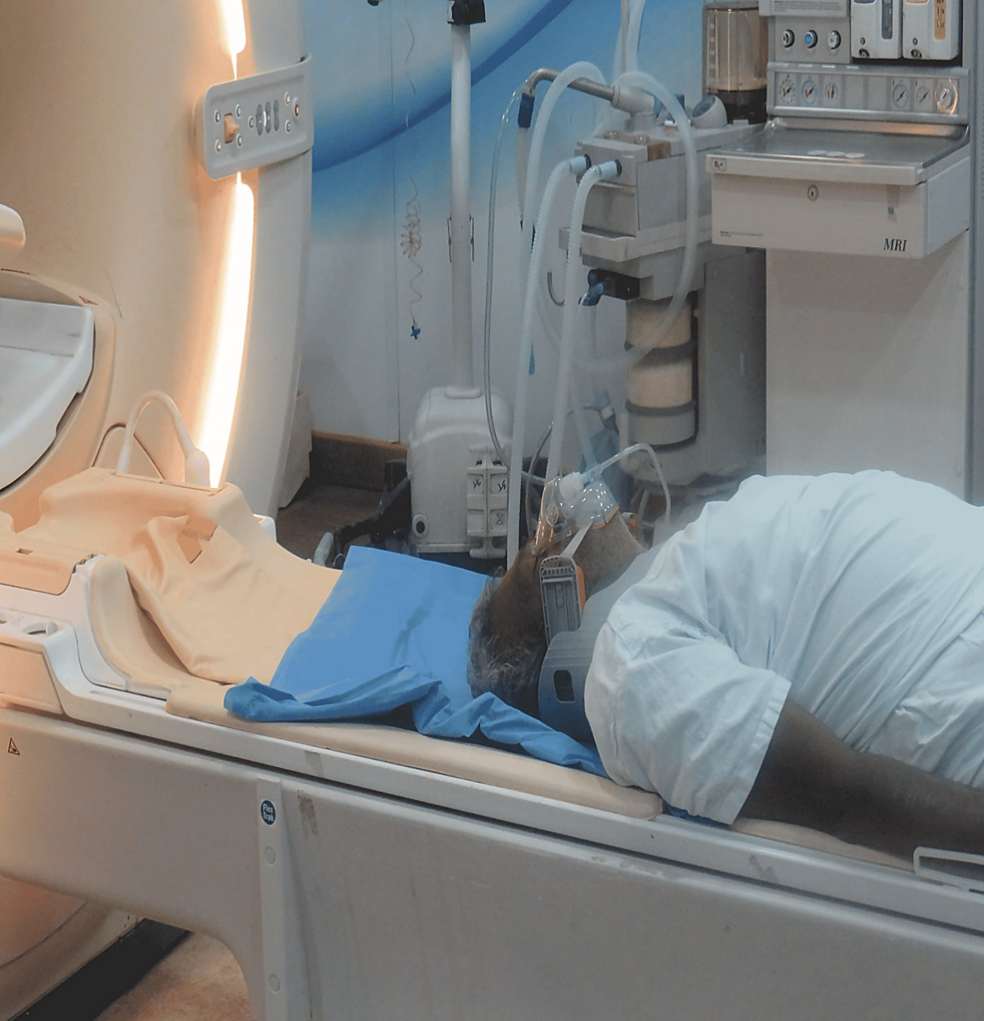
Lubo collar as a rescue during upper airway obstruction in the MRI suite.

A 45-year-old male (BMI: 33 kg/m^2^) diagnosed with chronic lumbar back pain was scheduled for an MRI of the lumbar spine. There was no associated medical comorbidity or any history of obstructive sleep apnea. Preoperative airway examination was unremarkable except for a mallampatti of class 3. As the patient was apprehensive and was unable to lie flat due to back pain, the MRI was planned under IV sedation after obtaining the patient consent. MRI-compatible ECG, peripheral pulse oximeter, and non-invasive blood pressure monitor (NIBP) were connected, and baseline vitals were recorded. The patient was supplemented with IV fentanyl of 50 mcg and 40 mg of IV propofol as a bolus. The sedation was maintained with IV propofol infusion at 75 mcg/kg/min through an MRI-compatible infusion pump Infusomat Space (B. Braun Medical, United States), titrated to hemodynamics and patient respiration. Oxygen was supplemented at 2 L/min through the nasal prongs, and the MRI was commenced. A slave monitor that was placed outside the MRI scanner room was used to monitor the vital parameters. The patient had a sudden drop in peripheral oxygen saturation (SpO2) to 85%, approximately 20 minutes following the commencement of the MRI. The MRI sequence was interrupted, and the patient was pulled out of the MRI gantry. Noisy labored breathing was noticed, suggestive of upper airway obstruction due to deep sedation, and the IV propofol was turned off immediately. Instead of a manual jaw thrust application, the Lubo collar was placed at the first response as it was readily available (Figure 1). The gliding knob of the collar was pulled forward to provide an automated jaw thrust to relieve the upper airway obstruction. After ensuring unobstructed and smooth breathing with the Lubo collar and normal SpO2 levels, the patient was taken back inside the MRI gantry. The MRI was continued with the Lubo collar in situ under propofol sedation at 50 mcg/kg/min that was reinstituted approximately 3 minutes after the event. At the end of the procedure, the IV propofol sedation was terminated, and the patient was awakened with no complications.

## Discussion

**Figure 2:**
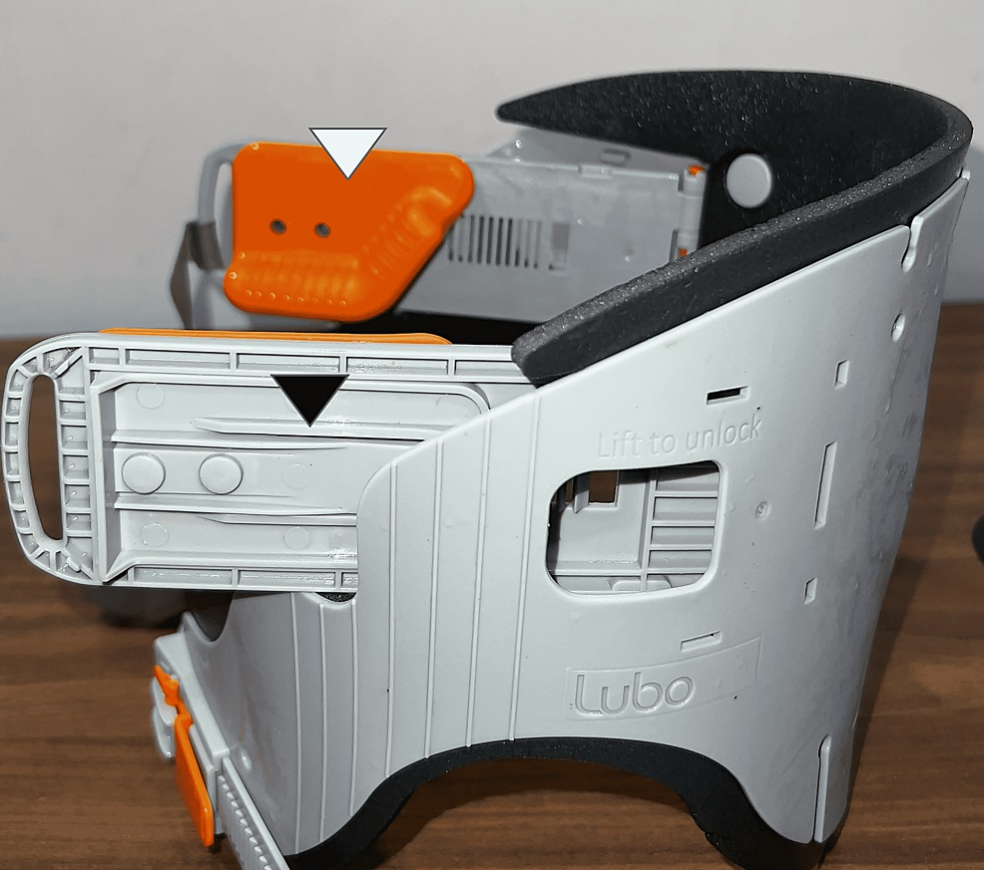
Lubo collar with mandibular rest (white arrowhead) and gliding knob (black arrowhead).

The management of an adverse respiratory event in the MRI suite can be challenging considering the lack of resources, manpower, and restricted access to the patient airway. The common tendency of the anesthesiologist is to provide a manual jaw thrust during an upper airway obstruction. As the Lubo collar was readily available within the MRI premises, we immediately used it as a rescue. The Lubo collar is simple to operate and provides an automated jaw thrust and cervical spine control simultaneously. It has a mandibular rest that fits at the angle of the mandible (Figure 2, white arrowhead) and a gliding knob (Figure 2, black arrowhead). Following the placement, the gliding knob of the collar can be pulled forward and locked, thereby simulating a manual jaw thrust. The act of forward pulling and locking the gliding knob in an awake patient can be painful. Hence its imperative to perform this following the administration of sedative agents. The Lubo collar can effectively maintain the upper airway patency in unconscious/anesthetized patients [2]. Vaithialingam B et al. have successfully utilized the Lubo collar to maintain the upper airway patency in conjunction with high flow nasal oxygen during the conduct of electroconvulsive therapy under general anesthesia [3]. The Lubo collar is MRI compatible, can be applicable at any head position, and also supports intubation with the collar in situ [4]. It also immobilizes the cervical spine through six touch points located outside the neck and has been used by paramedics in trauma services to support the cervical spine [4]. The applicability of the Lubo collar in a dystonic patient undergoing MRI has already been shown by Sundaram M et al. [5]. For patients with a high risk of airway obstruction like obstructive sleep apnea and morbid obesity, the Lubo collar can be placed electively following the administration of sedative agents, and the MRI can be performed with the collar in situ. We have successfully used this collar as a rescue in the MRI suite only after an adverse airway event that was accompanied by a rapid oxygen desaturation. These adverse events could have been avoided by titrating the IV sedation based on the depth of anesthesia monitors. Due to the non-availability of the MRI-compatible sensors, we could not perform depth sedation monitoring.

## Conclusions

The Lubo collar is MRI compatible and simple to operate and has a potential role as part of the airway management in the MRI suite. Even though we were successful with this novel airway collar, further clinical validation is needed in the future.
